# Use of Live Community Events on Facebook to Share Health and Clinical Research Information With a Minnesota Statewide Community: Exploratory Study

**DOI:** 10.2196/30973

**Published:** 2022-02-18

**Authors:** Jinhee Cha, Ian W West, Tabetha A Brockman, Miguel Valdez Soto, Joyce E Balls-Berry, Milton Eder, Christi A Patten, Elisia L Cohen

**Affiliations:** 1 Clinical and Translational Science Institute University of Minnesota Minneapolis, MN United States; 2 Center for Clinical and Translational Sciences Mayo Clinic Rochester, MN United States; 3 Knight Alzheimer's Disease Research Center Washington University in St. Louis St Louis, MO United States; 4 Department of Family Medicine and Community Health University of Minnesota Minneapolis, MN United States

**Keywords:** social media, virtual, digital, community engagement, engagement, retention, Facebook, health information, information sharing, communication, participation, retention, eHealth

## Abstract

**Background:**

Community engagement can make a substantial difference in health outcomes and strengthen the capacity to deal with disruptive public health events such as the COVID-19 pandemic. Social media platforms such as Facebook are a promising avenue to reach the broader public and enhance access to clinical and translational science, and require further evaluation from the scientific community.

**Objective:**

This study aims to describe the use of live community events to enhance communication about clinical and health research through a Facebook platform case study (*Minnesota [MN] Research Link*) with a Minnesota statewide community. We examined variables associated with video engagement including video length and type of posting.

**Methods:**

From June 2019 to February 2021, *MN Research Link* streamed 38 live community events on its public Facebook page, *MN Research Link*. Live community events highlighted different investigators’ clinical and health research in the areas of mental health, health and wellness, chronic diseases, and immunology/infectious diseases. Facebook analytics were used to determine the number of views, total minutes viewed, engagement metrics, and audience retention. An engagement rate was calculated by the total number of interactions (likes, shares, and comments) divided by the total length of the live event by the type of live community event.

**Results:**

The 38 live community events averaged 23 minutes and 1 second in duration. The total time viewed for all 38 videos was 10 hours, 44 minutes, and 40 seconds. Viewers’ watch time averaged 23 seconds of content per video. After adjusting for video length, promotional videos and research presentations had the highest engagement and retention rates. Events that included audience participation did not have higher retention rates compared to events without audience participation.

**Conclusions:**

The use of live community events showed adequate levels of engagement from participants. A view time of 23 seconds on average per video suggests that short informational videos engage viewers of clinical and translational science content. Live community events on Facebook can be an effective method of advancing health promotion and clinical and translational science content; however, certain types of events have more impact on engagement than others.

## Introduction

Community engagement is essential for advancing clinical and translational science (CTS) to improve public health [1]. Community engagement can make a substantial difference in health outcomes, strengthen the capacity to deal with disruptive public health events such as the COVID-19 pandemic, and empower communities [2]. In addition, community engagement that advances and disseminates CTS is critical to alleviating health disparities within marginalized communities and improving health outcomes [3,4]. Social media platforms such as Facebook provide a promising avenue to reach the broader public. The use of social media to access CTS content requires further evaluation by the scientific community [5].

Though Facebook is used as a platform for community engagement, the literature regarding its use to disseminate information about both research and health topics is still in its infancy [5,6]. Developing a Facebook community and using social networks to build engagement for health promotion and CTS content has been recognized as a valuable endeavor [7]. Evaluations of pre-existing Facebook communities involved with increasing community engagement and retention have been useful for future researchers interested in disseminating CTS content online [8-10]. In a previous program evaluation, our collaborative partnership between the Mayo Clinic (Clinic Center for Clinical and Translational Science [CCaTS]) and University of Minnesota’s Clinical and Translational Science Institute (CTSI) evaluated the feasibility of a virtual statewide Facebook community platform to enhance public engagement with health research in Minnesota [1]. Our partnership found that Facebook can be used as a community platform and is feasible in engaging Minnesotan residents in conversations around health and research topics. Nevertheless, additional research is needed to evaluate different types of Facebook posts and their potential to reach, engage, and retain community members with content posted on Facebook [1].

Facebook is one of the most widely used social media sites in the United States; 69% of adults report using Facebook, and its use is spread similarly across sociodemographic groups and geographic locations (urban/rural) [11]. For this reason, Facebook’s breadth and reach has made it particularly useful in reaching underserved or disadvantaged communities [12-14]. The use of Facebook in health interventions has demonstrated an improvement in participant retention and engagement with research studies [8,15]. For researchers, Facebook offers tools for measuring engagement from their audience through its built-in engagement metrics, including reactions (ie, *likes*), shares, or comments [16]. Many built-in Facebook capabilities such as livestreaming are used for public health promotion. For instance, Facebook Live allows users to schedule and then livestream broadcasts to a wide-ranging audience. Live streaming of health and education content can reach specific audiences through users’ social media routines and daily habits [8,17]. Therefore, Facebook, as a broad and established social media platform remains a viable tool for public health engagement that can be disseminated seamlessly into users’ social media routines and daily habits [8].

Learning about Facebook algorithms could assist researchers in building engagement. Facebook “pages” host information about an organization and broadcast updates to Facebook members based on unknown algorithms. On these pages, Facebook users have the option to create different types of posts and content (eg, status updates, videos, images, and live community events) [6]. Nevertheless, the algorithm dictates the amount of exposure an individual post may receive and, thus, controls the visibility of the Facebook page [6]. This algorithm is informed by an individual user’s unique interactions and behaviors with Facebook’s content [17]. Facebook incentivizes users to build virtual communities, maintain interests, and populate their “front page” on Facebook [18,19]. Though researchers operate without a broad understanding of Facebook’s proprietary algorithms (which can change without announcement), the literature suggests that certain types of content seem to gain preferential treatment. Posts and videos seem to gain more “likes'' and exposure in comparison to status updates [6,20]. Additionally, posts that garner more attention and interactions from users also seem to gain preferential treatment from Facebook’s algorithms. For instance, encouraging users to post feedback or asking the audience to participate with the page may encourage users to do so, leading to further dissemination on the platform [5].

Research examining types of social media posts and community engagement is in its infancy. Testimonials with positive emotional and informative posts are associated with higher engagement compared to negative emotional posts [6]. In an analysis among federal US health agencies’ Facebook posts, posts with more visual cues such as photos or videos, or those that express positive sentiment generated more engagement [21]. Evaluative studies of clinical research and health posts are not readily evident, although work on business posting assessing positive and negative posts found negatively valanced posts garnering more comments [22]. Recent discussions of *leaked* internal Facebook documents further suggest increased user exposure to *polarizing* content increases engagement with the platform [23]. Nevertheless, what is clear to social media strategists from these reports is that Facebook continues to be a widely used platform for content developers to engage with audiences as its news feed, advertisements, post likes, comments, and livestreaming opportunities continue to drive engagement. Since Facebook’s deployment of livestreaming, little research has investigated the role of live community events in fostering engagement or evaluating the use of livestreaming to promote health. The growth in its use has been in the field of strategic communication, and its impact to promote content has not yet been thoroughly investigated [24]. Thus, the question remains: how can researchers use the tools Facebook has to offer to create engaging content, use Facebook’s algorithms, and promote CTS content?

To address this question, a partnership between the Mayo Clinic CCaTS and University of Minnesota CTSI used Facebook’s tools to create the page *Minnesota (MN) Research Link*. *MN Research Link*’s mission was to provide credible information for Minnesota residents (1400 followers; n=434, 31% rural residents) in conversations about health and research topics, including health communication, health education, psychology, behavioral science, epidemiology, public health, and COVID-19 [1]. Content and posts on *MN Research Link* included status updates, videos, images (eg, research articles related to health research), and interactive postings/events (eg, live interviews with researchers and polls) [1].

To address the central research question, this study uses *MN Research Link* as a case to examine whether Minnesotans engage with live community events (ie, videos) on a Facebook page (*MN Research Link*) and whether live events can be used to build followers (audience) to support CTS. This case study serves to generate hypotheses for future researchers interested in using live community events on Facebook to generate community engagement and promote CTS content.

## Methods

### Study Platform

To examine how live community events can promote CTS content, a Facebook page, *MN Research Link*, was developed by the Mayo Clinic (CCaTS) and the University of Minnesota (CTSI) National Institutes of Health–funded CTS Community Engagement Programs. *MN Research Link* was created to serve as a community platform for public engagement on health research statewide in Minnesota [1]. The project was deemed exempt by both the Mayo Clinic and University of Minnesota institutional review boards.

#### Designing Live Community Events

Based on expert recommendations for disseminating CTS content, members of the research team constructed an internal calendar of live community events to broadcast on *MN Research Link* [3]. Experts were recruited from the University of Minnesota, the Mayo Clinic, and from local organizations who had experience in developing CTS content for social media. Based on their recommendations, specific health topics were featured on *MN Research Link* monthly. Members of the research team also met weekly to plan daily posts that were relevant to *MN Research Link*’s health topics and in accordance with the Centers for Disease Control and Prevention’s (CDC) social media guidelines [21]. Monthly health topics featured on *MN Research Link* included topics such as mental health, health and wellness (eg, nutrition and physical activity), chronic diseases (eg, cardiovascular disease), and immunology/infectious diseases. Over time, the research team adapted its monthly health topics to address important and relevant topics for followers. The final schedule included content on COVID-19, exercise, mental health, and well-being during the pandemic.

From June 2019 to February 2021 (20 months), four social media managers (authors JC, IWW, and MVS) facilitated 38 live community events on *MN Research Link*. Live community events of the following types were broadcasted on *MN Research Link* through the Facebook Live Event feature: brief interviews with experts, public health communications, research presentations, promotional videos for *MN Research Link*, and panels with expert public health professionals (Textbox 1). Through the Facebook Live Event feature and depending on the content and type of live community event, participants had the option of attending the live event through Zoom or watching on *MN Research Link*. During the live community events, social media managers facilitated discussions, moderated, or responded to participant questions on the *MN Research Link* page and through the Zoom chat. Guest speakers were recruited from the University of Minnesota, Mayo Clinic, and local organizations to participate in our live community events. When communicating with guest speakers, the social media managers emphasized that live community events were targeted for Minnesotans who may not have a background in science. Therefore, social media managers encouraged guest speakers to speak at a fifth-grade reading level.

**Key terms and their descriptions.**

**Interview**

A live event where a social media manager of *Minnesota (MN) Research Link* speaks with a presenter about their research and expertise over Facebook.

**Public health communication**

A live event where a social media manager of *MN Research Link* speaks about a public health subject.

**Research presentation**

A live event where a social media manager of *MN Research Link* attends a research conference and interviews poster presenters on their research.

**Promotional video**

A live event where a social media manager of *MN Research Link* promotes *MN Research Link* events and posts or encourages engagement.

**Panel**

A live event where a social media manager of *MN Research Link* facilitates a conversation with more than one presenter on a public health subject.

**Audience participation**

A feature of live community events where a social media manager of *MN Research Link* encourages viewers to engage with the event by asking questions in the chat, asking questions live, or asking for feedback on the event.

**Reactions (likes)**

An engagement metric from Facebook Insights. User-generated indicators of post engagement that includes “like,” “love,” “haha,” “wow,” “sad,” and “angry.”

**Shares**

An engagement metric from Facebook Insights. Additionally a feature of Facebook posts where users may share the post/content on their own social media page and groups or with other users.

**Comments**

An engagement metric from Facebook Insights. User-generated comments, including replies to an individual post. Does not include comments from shares.

**Clicks**

An engagement metric from Facebook Insights. The number of unique clicks a user has made to play a live event.

**Audience retention**

An engagement metric from Facebook Insights. The average length of time viewers watch a video.

#### Promotion of Live Community Events

For interviews and panels, promotional materials were posted on *MN Research Link* at least 1 week before the date of the live event. Promotion materials included a post featuring graphics with details of the events, details on how to join the live event, and a promotion video from one of our social media managers briefly talking about the event. To create promotional videos, a research assistant uploaded a brief video (around 2 minutes) explaining the event, introducing the guest speaker, and sharing details of the live community events at least 2 days prior to the live event. Before a live event, approximately 1.5 hours were spent between the meetings communicating with the presenter or presenters, creating relevant posts, and creating a promotional video.

Advertisements or targeted “boosts” (Facebook’s term for creating ads for posts across Facebook pages) [16] were used to expand the reach of the page to new audiences and to promote attendance for the live community events among Minnesotan Facebook users 18 years or older. Posts on the platform that receive paid boosts demonstrate greater reach and engagement with new audiences than posts that do not [22] and are an appropriate use of research funds to enhance audience reach. Approximately one-third of events were boosted 1 week prior to their start date. We used boosts to attract new followers and did not conceptualize boosts as a direct way to increase live event–specific engagement or retention rates. Boosts for *MN Research Link* posts occurred approximately 5 to 7 days prior to the event and were targeted for Minnesotan Facebook users 18 years and older. University of Minnesota and Mayo Clinic staff members, researchers, stakeholders, and community partners were asked to share events hosted by the *MN Research Link* page on their organization’s Facebook page. Additionally, these members also shared events with individuals within their social network.

#### Procedure

Ideas for live community events were generated by weekly meetings between the Mayo Clinic CCaTS and University of Minnesota CTSI based on monthly health topics. After generating ideas for live community events, professors and researchers at the University of Minnesota and Mayo Clinic were invited as subject matter experts to participate in the live community events. For instance, when the health topic was “exercise during COVID-19,” we invited a university presenter to talk about her research on exercise and how to exercise during the pandemic. Invitations were sent by email at least 2 weeks prior to the suggested live event date with information about presenters and the project. Additionally, we encouraged presenters to share their research, in any form, ahead of time on *MN Research Link*. For instance, for the live event planned on “exercise during COVID-19,” the presenter provided exercise science research to share on *MN Research Link*. Before the live event, the social media manager facilitated the live event to make sure the technology was working properly and that the presenter was ready. The length of the live events differed by type; interviews aimed to last less than 15 minutes in duration, whereas public health communications, research presentations, and promotional videos for *MN Research Link* were shorter (some less than 5 minutes in duration). Panels featuring expert public health professionals were less than an hour long.

#### Engagement Metrics and Analysis

To examine how live community events on *MN Research Link* can promote CTS content, a variety of metrics were used to measure audience engagement. Broadly considered, engagement metrics are metrics such as the number of followers, “likes,” shares, clicks, and comments on individual live community events (Textbox 1). Although Facebook includes other reactions aside from “likes” (eg, “love,” “care,” or “laugh”) as part of its engagement reaction metrics, for the purposes of our study, we operationalized all of these types of reactions to posts as a part of a singular Facebook engagement metric (Textbox 1).

The study used engagement metrics produced using Facebook Insights. Facebook Insights provides quantitative information of engagement metrics for all posts and content on a Facebook page [16]. Facebook Insights were used to generate descriptive statistics per live community events such as the total minutes viewed and audience retention. The audience retention rate was defined by Facebook as the average length of time (in seconds) viewers watch a video (Textbox 1). We also formulated our own “engagement score” by summarizing the total number of reactions (likes, shares, and comments) with an individual post (Textbox 1). The engagement score was used to summarize the reach and participation with *MN Research Link* through June 2019 to February 2021 (20 months) when live community events were implemented. Certain types of live community events may attract more attention than others because of Facebook’s algorithms [18]. Moreover, longer live events offer more opportunities to engage with the content during the live broadcast. Therefore, we controlled for video length to determine the engagement and retention by the type of live community event.

To examine how audience participation on *MN Research Link* can promote CTS content, we compared engagement rate and audience retention rate across live community events with and without audience participation. We defined events with audience participation as events that featured the audience participating with the chat, asking questions live, or asking for feedback on the event. For example, for a panel on “Mental Health during COVID-19,” the social media managers encouraged the audience to ask questions or make comments in the chat.

## Results

Key terms and their descriptions are included in Textbox 1. A total of 38 live community events were hosted by *MN Research Link* using the Facebook platform (Table 1). Events averaged 23 minutes and 1 second. The total time viewed for all 38 videos was 10 hours, 44 minutes, and 40 seconds. Viewers spent an average of 23 seconds watching each piece of video content (Table 1).

**Table 1:** Descriptive statistics for all live video events, June 2019 to November 2020 (n=38).

Descriptive statistic	Value
Video length (hh:mm:ss), mean	00:23:01
Total length of video viewed (hh:mm:ss)	10:44:40
Audience retention (hh:mm:ss), mean	00:00:23

Interviews were the most frequent type of live community event hosted on *MN Research Link* (n=19; total video length 5:51:39), whereas promotional videos and panels were the least frequent type of live community events (n=3 and n=2; total video length 00:05:43 and 00:05:54, respectively; Table 2). After controlling for video length, promotional videos had the highest average engagement rate (0.404), followed by research presentation (0.224). After adjusting for video length, promotional videos and research presentations had the highest retention rate (0.096 and 0.090, respectively). Panels had the lowest engagement and retention rate, after adjusting for video length (0.001 and 0.020, respectively; Table 2).

**Table 2:** Types of live community events and engagement metrics adjusted by video lengths.

Type of event	Events, n (%)	Video length (hh:mm:ss)	Engagement rate^a^	Retention rate^b^
Interview	19 (50)	05:51:39	0.045	0.021
Public health communication	10 (26)	01:30:06	0.017	0.040
Research presentation	4 (11)	00:05:43	0.224	0.090
Promotional video	3 (8)	00:05:54	0.404	0.096
Panel	2 (5)	01:53:40	0.001	0.020

^a^Engagement rate is calculated by the sum engagement divided by the sum video length in seconds.

^b^Retention rate is calculated by the sum retention divided by the sum video length in seconds.

Of 38 live community events, 29 of them did not ask for or include audience participation in terms of questions and answers (Table 3). However, event engagement rates between events with audience participation and events without audience participation looked similar (0.042 and 0.049, respectively; Table 3). In contrast, events that did not include audience participation had higher audience retention rate scores compared to events with no audience participation (0.026 and 0.016, respectively; Table 3).

**Table 3:** Audience participation and engagement metrics adjusted by video length in seconds.

Audience participation type	Events, n (%)	Video length (hh:mm:ss)	Engagement rate^a^	Retention rate^b^
No audience participation included	29 (76)	07:27:28	0.042	0.026
Audience participation included	9 (24)	03:17:12	0.049	0.016

^a^Engagement rate is calculated by the sum engagement divided by the sum video length in seconds.

^b^Retention rate is calculated by the sum retention divided by the sum video length in seconds.

## Discussion

### Principal Results

To our knowledge, our study is the first to examine the use of live community events over Facebook to promote CTS content through community engagement. This case study examined whether Minnesotans would engage with live community events (ie, live videos) on a Facebook page (*MN Research Link*) and whether live community events could be used to build followers (audience) to support CTS. Using a novel engagement metric and tools by Facebook Insights, we summarized both the reach and engagement with followers on *MN Research Link*. Understanding how live community events can engage the public and how audience participation may enhance the promotion of CTS content (both in terms of their reactions to it and its stick-with-it-ness) will better inform researchers on how to reach communities via social media [20].

The findings indicate that live community events engaged users for CTS content. Facebook reports that the average retention for videos is 16.7 seconds [25], whereas the average retention for live community events on *MN Research Link* was 23 seconds (Table 1). Our retention time suggests that live community events are a better medium for retention than regular videos.

“Likes” or “reactions” to posts on Facebook suggests that users appreciated, liked, or were impacted with the post more so than usual [13,14]. The engagement rate score summed all reactions on each post to identify which types of CTS content garnered the most community engagement. The findings suggest that certain types of live community events have more of an impact on engagement than others; promotional videos (which generally contain minimal CTS research content) had the highest engagement, followed by research presentations, whereas public health communications and panels had the lowest amount of engagement (Table 3). Similarly, promotional videos had the highest retention rate, followed by research presentations. This suggests that social media users from Minnesota, on average, engaged with promotional videos the most and stayed engaged for longer periods of time compared to other types of live community events.

Generally, events with no audience participation garnered more retention for live community events but had minimal impact on engagement. More specifically, live community events with no audience participation element had 1.63 times the retention metric compared to live community events with audience participation (Table 3). This suggests that including audience participation does not engage social media users through “likes,” “reactions,” or “shares” and may even decrease how long audience members may remain attentive toward live community events. We offer two possible explanations as to why retention was lower for live community events with audience participation. First, audience participation requires more effort; viewers may not want to remain attentive to an event if they are asked to participate. Additionally, live community events with audience participation included panels that were significantly longer than other types of content. Since average retention was calculated by sum retention divided by sum video length in seconds, having longer videos would increase the denominator, resulting in a smaller quotient.

### Implications

Our results add to the literature by suggesting that live community events on Facebook can not only be an effective method of advancing CTS content but also be better than conventional posts with just pictures or videos. Our research builds on prior research suggesting the use of photos and videos garner more community engagement compared to posts that do not include photos and videos [6]. It suggests that though video engagement is important, the nature of the content matters for engagement, and researchers should consider the length of videos posted for users for CTS content promotion.

Developing and promoting live community events was a time intensive process; planning for a live event alone took approximately 1.5 hours. However, our case study demonstrates these events were only watched for an average of 23 seconds. This presents researchers with an interesting challenge; condensing CTS information into 30 seconds or less may be difficult for research dissemination. Ultimately, short form live community events may be more cost- and time-effective compared to longer videos for audience retention and engagement [25]. Ensuring viewers are greeted with a story and getting straight to the point, even if it is short, may also result in greater engagement and retention for the video [25]. Additionally, when hosting a longer live community event, asking viewers to participate may not result in increased retention. Thus, these findings raise appreciable evidence of interest for medical researchers and public health promotion experts seeking to leverage Facebook and other social media to improve audience engagement.

Findings from this case study confirm related findings on how brief informational video interventions, particularly those that are interactive and engaging, play important roles in health promotion. Data from this case study also suggests that short video content (eg, TikTok length) may be repurposed for effective use on Facebook to engage with the audience. This confirms the CDC’s 2018 Health Communication Playbook, which identifies this strategy for consumer communication: keep information short and concise, avoid jargon, and use a content calendar to strategically engage your audience [26].

### Conclusions

Although this case study demonstrates the ways that the *MN Research Link* page engages with its audience, the metrics provided are limited with respect to the constraints of the platform [16]. Future research could also evaluate how different health topics engage or retain Facebook users for CTS content. For example, because likes and shares are public [14], posts with stigmatizing topics such as mental health may or may not gain engagement compared to less stigmatizing topics. Such research could also examine how short form live community events or videos can be time-effective and offer more condensed information all while retaining audience retention. More work is needed, however, to determine how CTS content can be promoted through live community events though other social media networks (eg, Instagram Live or Twitch). These findings emphasize the need for ongoing CTS promotional research to ensure that accessible relevant health information is readily available for all social media users [2,12,27].

## Abbreviations

CCaTS: Clinic Center for Clinical and Translational Science
CDC: Centers for Disease Control and Prevention
CTS: clinical and translational science
CTSI: Clinical and Translational Science Institute
MN: Minnesota
